# Can cervical spine axial GRE imaging replace FSE T2 axial imaging in the evaluation of central canal stenosis?

**DOI:** 10.1007/s00234-026-03998-7

**Published:** 2026-06-13

**Authors:** Jinyoung Choi, Hee Jin Park, Ji Na Kim, Myung Sub Kim

**Affiliations:** 1 Kangbuk Samsung Hospital, Sungkyunkwan University, School of Medicine, Seoul, Korea, Republic of; 2 Chung-Ang University Hospital, Chung-Ang University College of Medicine, Seoul, Korea, Republic of

**Keywords:** Central cervical spinal stenosis, Magnetic resonance imaging, Cervical spine

## Abstract

**Background:**

Many hospitals perform both axial fast spin-echo T2-weighted image (FSE T2WI) and axial gradient recalled echo T2-weighted (GRE) imaging.

**Purpose:**

This study was performed to compare inter-reader agreement of cervical central spinal stenosis (CCSS) grading with the use of axial GRE imaging and axial FSE T2WI. We also compared the correlations between each radiologic grade and clinical manifestations.

**Methods:**

A total of 143 patients (M: F = 71:72; mean age, 52 years) who underwent magnetic resonance imaging of the cervical spine at our hospital were included. Two radiologists evaluated the degree of CCSS from the level of C2–3 to the level of C6–7 using axial GRE imaging, axial FSE T2WI, axial GRE imaging with sagittal T2WI, and axial FSE T2WI with sagittal T2WI. Kappa statistics were used to analyze the inter-reader agreement.

**Results:**

The existence of substantial agreement between GRE axial and FSE T2 axial images was reported by both readers (0.657 ≤ κ ≤ 0.665), who also reported almost-perfect agreement between GRE axial + T2 sagittal and T2 axial + T2 sagittal imaging (0.958 ≤ κ ≤ 0.979). The GRE axial, GRE axial + T2 sagittal, and T2 axial + T2 sagittal images showed superior correlation (moderate) compared to that of T2 axial images only (weak) in revealing the correlation between Kang grade and clinical manifestation.

**Conclusion:**

The agreement of CCSS grading with axial GRE imaging and axial FSE T2WI findings was substantial. Using axial GRE images led to a superior correlation between magnetic resonance sequence and clinical manifestations relative to using axial FSE T2WI.

## Introduction

Cervical spinal stenosis is a common degenerative condition characterized by the narrowing of the spinal canal, which can potentially lead to cervical spondylotic myelopathy (CSM), particularly in elderly patients. Narrowing of the spinal canal may be caused by various factors, including the herniation or bulging of intervertebral disks, osteophytes, and ossification of the posterior longitudinal ligament [1]. Cervical central spinal stenosis (CCSS) refers to a narrowing of the spinal canal. As a result, the progressive compression of the cord by these factors may lead to spinal cord ischemia, resulting in histopathologic changes of the cervical spinal cord [2, 3]. While CCSS is often a chronic process, acute or progressive cord compression requires prompt diagnosis to prevent irreversible spinal cord injury [4]. The diagnosis of CCSS is made using the clinical history; by assessing neurological signs; and by imaging studies, including plain radiography, computed tomography (CT), myelography, and magnetic resonance imaging (MRI). MRI is the gold standard for CCSS imaging, offering superior soft-tissue contrast compared to CT, which allows for better visualization of the spinal cord, cerebrospinal fluid space, and soft tissue structures [5]. Generally, it is important to assess the degree of canal stenosis to facilitate better management of patients. There is no universally accepted grading system for CCSS, though some studies have reported various methods of qualifying or quantifying the degree of cervical stenosis [6].

To date, some studies have reported inconsistencies in predicting clinical outcomes in patients with CCSS [7–9]. Kang’s grading system is a novel MRI grading system for CCSS, and we researchers have published several correlation studies about the usefulness of this system to date [1, 4, 6]. In their seminal research, Kang et al. classified CCSS into 4 grades. It has been proven that the Kang system is a very reliable method for grading CCSS [1, 4, 6] with near-perfect inter-observer agreement and a significant correlation with neurologic manifestations [4, 6]. The success of Kang grading mainly relies on sagittal T2-weighted imaging (T2WI); however, in the case of uncertain stenosis, readers could refer instead to axial T2WI to clarify the degree of stenosis [4, 6].

A sequence of gradient recalled echo (GRE) T2WI acquires a gradient echo signal. The MRI signal is extremely sensitive to motion, and pulsatile movements of the cerebrospinal fluid are known to cause artifacts on cervical spine images in the forms of signal losses and phase shifts, which can largely be eliminated by using a GRE sequence [10]. GRE imaging uses lower flip angles than spin echo sequences, resulting in a lower signal-to-noise ratio (SNR), a higher risk of magnetic susceptibility artifacts, and lower image resolution, whereas fast spin echo imaging (FSE) offers higher SNR and reduced magnetic susceptibility artifacts [11]. For this reason, many hospitals perform both axial FSE T2WI and axial GRE imaging, but the total scan time is necessarily very long as a result. If axial GRE imaging demonstrates comparable reliability to axial FSE T2WI in evaluating CCSS, the scan time can be greatly reduced by adopting only the GRE sequence in the evaluation of CCSS. The hypothesis of this study was therefore that axial GRE images had similar inter-reader concordance and correlation coefficients as axial FSE T2WIs in the assessment of CCSS.

The purpose of this study was to compare inter-reader agreement of CCSS grading with the use of axial GRE and axial FSE T2WIs. We also compared the correlations between each radiologic grade and clinical manifestations.

## Methods

### Case selection

This retrospective study was performed in accordance with the Declaration of Helsinki. Of the patients who underwent cervical spine MRI at our hospital between December 2015 and September 2020, 171 consecutive patients were selected for CCSS assessment. The clinical indications for cervical spine MRI were persistent posterior neck pain, radiating pain to the bilateral or unilateral upper extremities, weakness of the fingers or hand, and tingling or numbness of the upper extremities. We excluded 28 patients to get rid of confounding factors for the following reasons, respectively: multilevel stenosis or cervical neural foraminal stenosis (20 cases), history of acute trauma (3 cases), and previous surgical history (5 cases). Patients with multilevel central stenosis or combined cervical neural foraminal stenosis were excluded because correlating a single imaging grade with focal clinical manifestations is challenging in such cases. Exclusion criteria were determined by review of imaging findings by the study readers rather than by radiology reports alone. And we also excluded other conditions that could cause paresthesia, tingling, pain, and weakness in the upper extremities, such as peripheral neuropathy changes secondary to metabolic conditions, vitamin deficiencies, diabetes, chemotherapy, and other causes. Ultimately, 143 patients were selected for analysis. The final study group consisted of 71 men and 72 women (average age, 51.69 ± 14.82 years; age range, 23–86 years).

### Clinical correlation

A neurosurgeon with 24 years of experience in spine surgery assessed the clinical data, including patients’ histories, symptoms, and neurological examinations, based on the content of electronic medical records. Positive clinical manifestations (PCMs) were defined as the following symptoms or signs in patients: paresthesia, extremity weakness, numbness, and funicular or radicular pain. Positive neurologic signs included a positive Lhermitte sign, Spurling sign, increased response of the deep tendon reflex, and a positive denervation sign on electromyography [6]. Relief of clinical symptoms after surgery or nerve block was also considered a PCM of central spinal stenosis.

### MRI parameters

All MRI scans for evaluating the cervical spine in our hospital were performed using a 1.5-Tesla scanner (Intera; Philips Healthcare, Best, The Netherlands) with a Syn-head coil (Philips Healthcare). A head coil was used because it provided sufficient signal coverage of the upper cervical spine with acceptable image quality in our routine clinical protocol. Axial and sagittal MRI scans were obtained with the patient in the supine position. Three-dimensional FSE sequences were not routinely included in our protocol during the study period and therefore were not evaluated in this retrospective analysis. The imaging parameters for the different imaging techniques are listed as follows: axial T2-weighted fast spin-echo (FOV = 150 mm, TR/TE = 2500/100, matrix = 250 × 180, slice thickness = 3 mm, inter-slice gap = 0.3 mm, signal average = 1, scan time = 1 min 20 s), GRE axial imaging (FOV = 150 mm, TR/TE = 400/5, flip angle = 25, matrix = 150 × 150, slice thickness = 3 mm, inter-slice gap = 0.3 mm, signal average = 1, scan time = 1 min 30 s), sagittal T2-weighted spin-echo (FOV = 220 mm, TR/TE = 3000/100, matrix = 220 × 220 matrix, slice thickness = 3 mm, inter-slice gap = 0.3 mm, signal average = 1, scan time = 2 min 40 s ), axial T1-weighted spin-echo (FOV = 150 mm, TR/TE = 600/15, matrix = 330 × 250, slice thickness = 3 mm, inter-slice gap = 0.3 mm, signal average = 1, scan time = 1 min 50 s), and sagittal T1-weighted spin-echo (FOV = 220 mm, TR/TE = 700/10, matrix = 450 × 200, slice thickness = 3 mm, inter-slice gap = 0.3 mm, signal average = 1, scan time = 2 min 20 s). Total scan time was 9 min 40 s.

### Image analysis

**Fig. 1:**
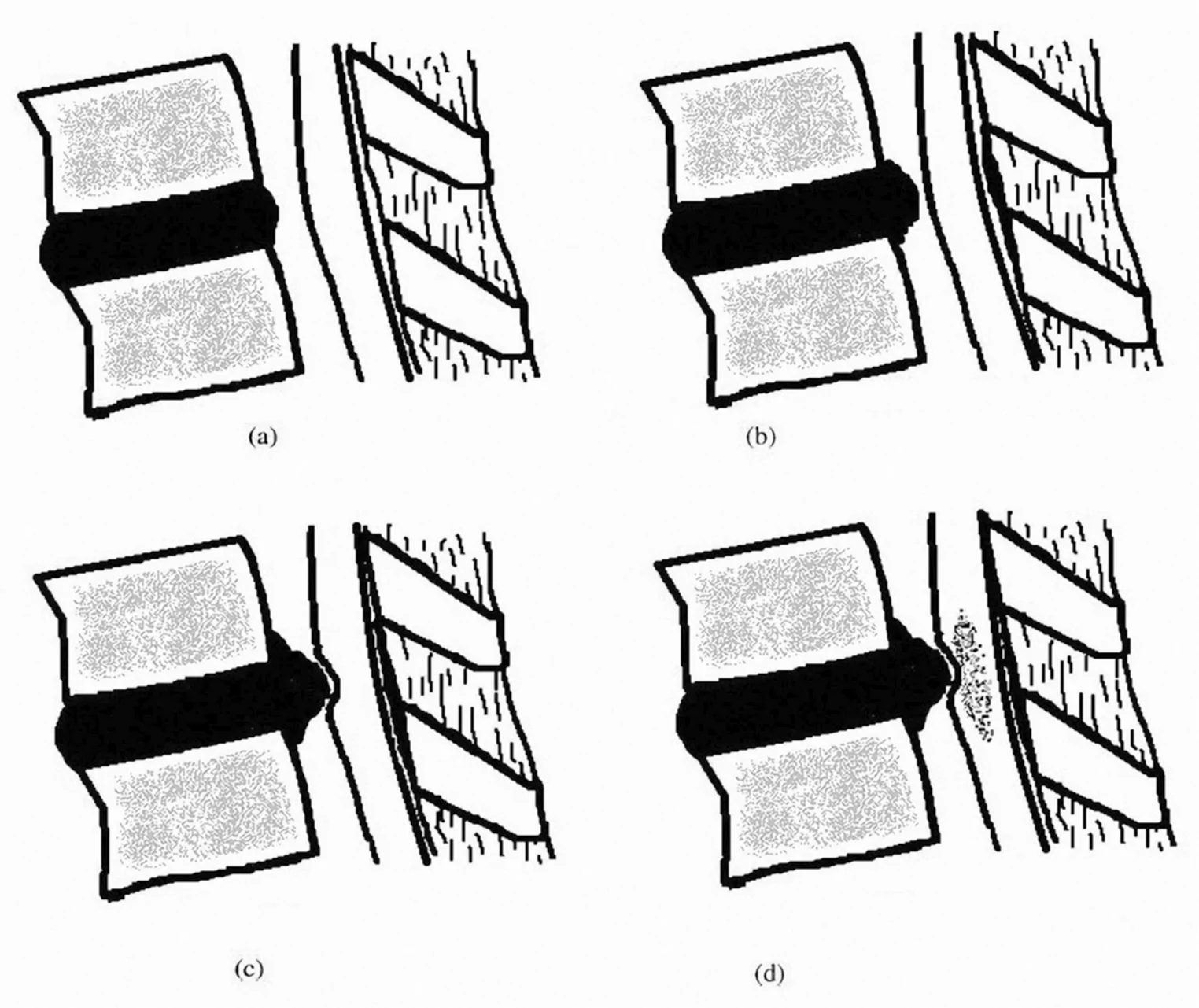
Schematic diagrams of Kang’s grading system of cervical canal stenosis based on the mid-sagittal plane. (adapted from the work of Lee et al. with permission). **A**. Grade 0, obliteration of the subarachnoid space < 50%. **B**. Grade 1, obliteration of the subarachnoid space ≥ 50% without a demonstrably deformed cord. **C**. Grade 2, demonstrably deformed cord without cord signal change at the level of deformity. **D**. Grade 3, spinal cord compression with signal change at the level of compression.

**Fig. 2:**
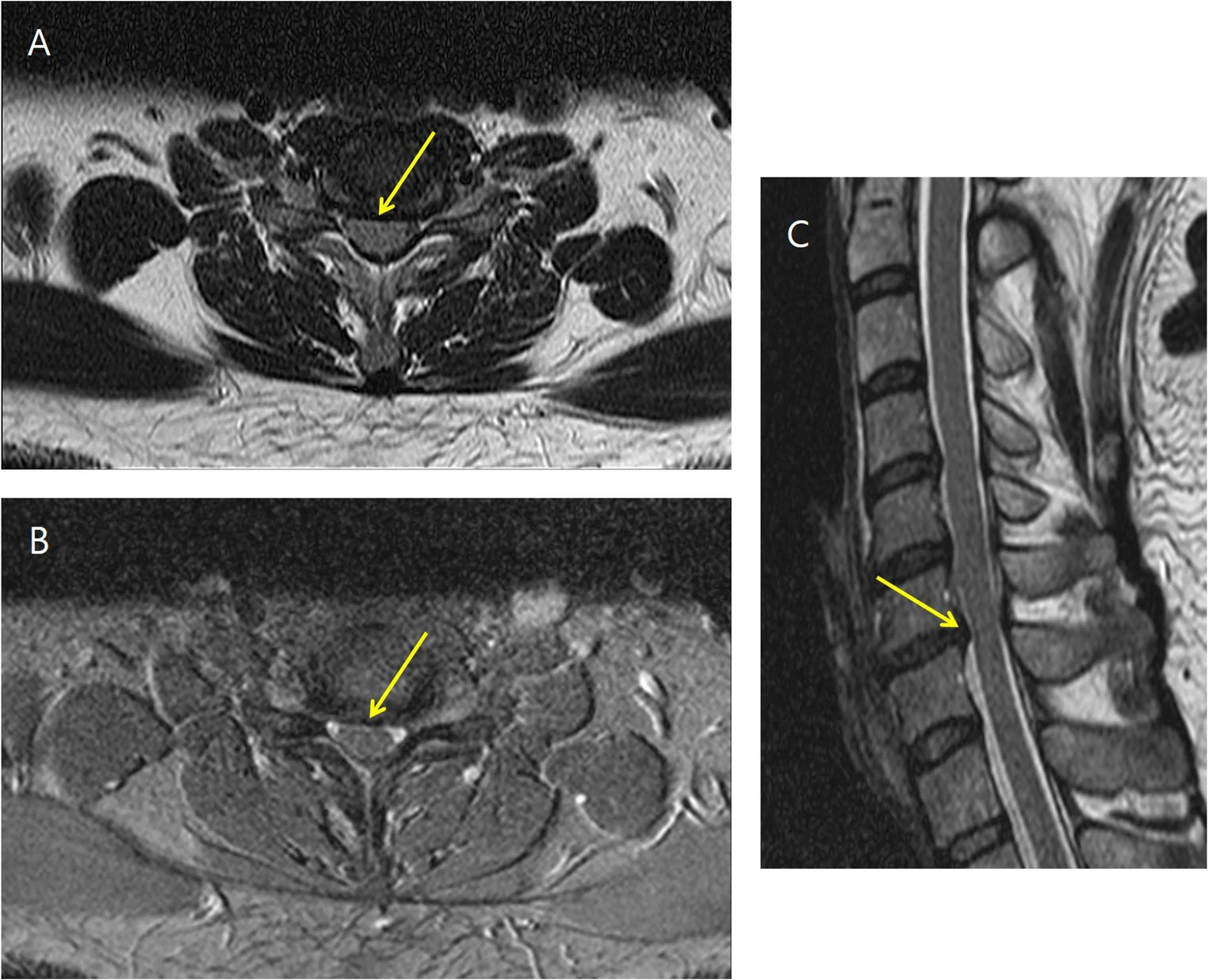
Example of a Kang grade 2 case involving 50-year-old man with diffuse disc bulging at the C6–7 level. A, B. T2 axial image (**A**) and GRE axial image (**B**) show a grade 2 central spinal stenosis case with slightly deformed spinal cord caused by a diffuse bulging disc at the C6–7 level. C. T2 sagittal image shows that the diffusely bulging disc is compressing the spinal cord without definite signal change.

MRI scans were interpreted by a pair of fellowship-trained academic radiologists with 20 and 10 years of experience of neuroimaging, respectively. Both readers were blinded to patient clinical information and radiologic reports. Before completing their assessment, these readers read an article about the Kang system and underwent training sessions lasting several hours. As stated previously, Kang et al. classified CCSS into 4 grades (Fig. 1), including grade 0, subarachnoid space obliteration < 50% (absence of canal stenosis); grade 1, subarachnoid space obliteration > 50%; grade 2, compressed spinal cord without signal change in the cord; and grade 3, spinal cord compression with signal change [1, 4]. They evaluated the degree of CCSS from the level of C2–3 to the level of C6–7 using axial GRE images only. Then, 3 weeks later, the readers evaluated the degree of CCSS based on axial FSE T2WIs only. After another 3 weeks, they evaluated patients based on axial GRE and sagittal T2WI images. Finally, after another 3 weeks, they evaluated patients using FSE T2WI and sagittal T2WIs again (Fig. 2).

### Statistical analysis

Inter-observer agreement between the pair of radiologists was analyzed by MRI sequences using kappa (κ) statistics. The strength of the inter-observer agreement was interpreted as follows: poor (κ < 0.1), slight (0.1 ≤ κ ≤ 0.2), fair (0.2 < κ ≤ 0.4), moderate (0.4 < κ ≤ 0.6), substantial (0.6 < κ ≤ 0.8), and nearly perfect (0.8 < κ ≤ 1) [12]. The correlation coefficient (Rs) was assessed using Spearman’s correlation statistic, and the relationship between clinical symptoms and CCSS grade was analyzed as either a weak correlation, 0.1 < *R* ≤ 0.3; a moderate correlation, 0.3 < *R* ≤ 0.7; a relatively high correlation, 0.7 < *R* ≤ 0.9; or a very high correlation, 0.9 < R. In all cases, the correlation was significant at the 0.01 level [13] and *P* ≤ 0.05 was considered to be statistically significant. Statistical analyses were performed using PASW software version 18.0 (IBM Corporation, Armonk, NY, USA).

## Results

**Fig. 3:**
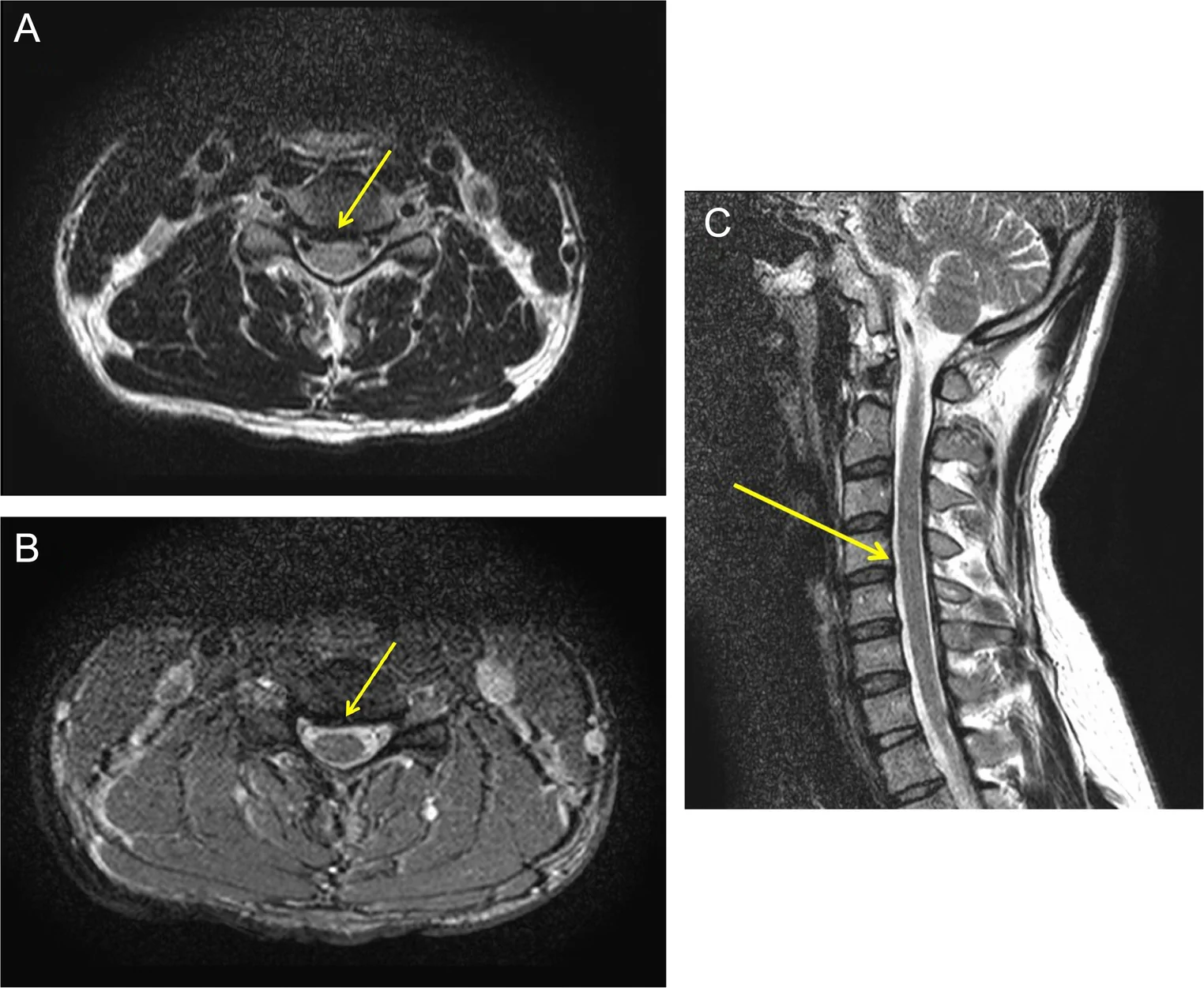
Example of a Kang grade 0 case in a 29-year-old man. A. T2 axial image. The T2 high signal intensity in the subarachnoid space is almost invisible at the C4–5 level, so it was determined to be grade 1 central spinal stenosis. B. GRE axial image shows that the subarachnoid space with high signal intensity > 50%. The Kang grade was 0. C. T2 sagittal image shows that there is no obvious abnormality in the subarachnoid space at the C4–5 level. The Kang grade was 0. Therefore, the signal void lesion seen on the T2 axial image was a flow artifact.

The prevalence of Kang grades according to the MRI sequence and the PCM incidence of each MRI grade are shown in Table 1. Although there are some exceptions realized by reader and by sequence combination, Kang grade 2 showed the highest prevalence, followed by grades 1, 0, and 3, respectively. PCMs were found in 33%–40% of all grade 0 patients, 62%–73% of all grade 1 patients, 64%–69% of all grade 2 patients, and 83%–100% of all grade 3 patients, respectively. Table 2 depicts the inter-observer reliability evaluated by κ statistics; notably, there was almost-perfect agreement between both readers on every combination of sequences (0.937 ≤ κ). Table 3 shows the intra-observer reliability between GRE axial and T2 axial images and between GRE axial + T2 sagittal and T2 axial + T2 sagittal images, respectively. Both readers reported substantial agreement regarding the correlation between GRE axial and T2 axial images (0.657 ≤ κ ≤ 0.665) and almost-perfect agreement regarding the correlation between GRE axial + T2 sagittal and T2 axial + T2 sagittal images (0.958 ≤ κ). When grading using GRE axial or T2 axial sequences alone, reader 1 yielded 111 matches and 32 mismatches and reader 2 yielded 109 matches and 34 mismatches out of 143 total matches, respectively; however, the difference in grades was only 1 step (i.e., grade 1 vs. grade 2). When grading using GRE axial with sagittal images or T2 axial with sagittal images, reader 1 yielded 2 mismatches and reader 2 yielded 4 mismatches out of 143 total matches, respectively, with the difference in grades also being just a single step. In an example of a mismatch, the patient had a grade 2 central stenosis as judged by the T2 axial image but appeared to have a grade 1 stenosis on the GRE image. (Fig. 3)

**Table 1:** Prevalence of Kang grade according to the MRI sequence and the incidence of positive clinical manifestations of each MRI grade

Reader Sequence	Parameter	Kang grade			
		0	1	2	3
Reader 1 GRE axial	Prevalence	24 (16.8%)	46 (32.2%)	58 (40.6%)	15 (10.5%)
	Positive clinical manifestations	8 (33.3%)	33 (71.7%)	39 (67.2%)	13 (86.7%)
Reader 2 GRE axial	Prevalence	24 (16.8%)	44 (30.8%)	58 (40.6%)	17 (11.9%)
	Positive clinical manifestations	8 (33.3%)	32 (72.7%)	39 (67.2%)	14 (83.4%)
Reader 1 T2 axial	Prevalence	9 (6.3%)	66 (46.2%)	59 (41.3%)	9 (6.3%)
	Positive clinical manifestations	3 (33.3%)	43 (65.2%)	38 (64.4%)	9 (100%)
Reader 2 T2 axial	Prevalence	9 (6.3%)	65 (45.5%)	59 (41.3%)	10 (7.0%)
	Positive clinical manifestations	3 (33.3%)	41 (63.1%)	39 (66.1%)	10 (100%)
Reader 1 GRE axial + T2 sagittal	Prevalence	21 (14.7%)	49 (34.3%)	61 (42.7%)	12 (8.4%)
	Positive clinical manifestations	8 (38.1%)	31 (63.3%)	42 (68.9%)	12 (100%)
Reader 2 GRE axial + T2 sagittal	Prevalence	22 (15.4%)	42 (29.4%)	66 (46.2%)	13 (9.1%)
	Positive clinical manifestations	8 (36.4%)	29 (69.0%)	44 (66.7%)	12 (92.3%)
Reader 1 T2 axial + T2 sagittal	Prevalence	20 (14.0%)	50 (35.0%)	62 (43.4%)	11 (7.7%)
	Positive clinical manifestations	8 (40.0%)	31 (62.0%)	43 (69.4%)	11 (100%)
Reader 2 T2 axial + T2 sagittal	Prevalence	21 (14.7%)	42 (29.4%)	69 (48.3%)	11 (7.7%)
	Positive clinical manifestations	8 (38.1%)	28 (66.7%)	46 (66.7%)	11 (100%)

* Data: Numbers in parentheses, in the prevalence row, represent percentages of the total number

** Numbers in parentheses in the positive clinical manifestations row are percentages of the total number for the corresponding grade

**Table 2:** Inter-observer reliabilities between readers

	GRE axial	T2 axial	GRE axial + T2 sagittal	T2 axial + T2 sagittal
Reader 1 vs. reader 2	0.970 (0.936-1.000)	0.954 (0.910–0.999)	0.938 (0.888–0.987)	0.937 (0.886–0.987)

* Values are given as kappa (κ) values: poor (κ < 0.1), slight (0.1 ≤ κ ≤ 0.2), fair (0.2 < κ ≤ 0.4), moderate (0.4 < κ ≤ 0.6), substantial (0.6 < κ ≤ 0.8), and almost perfect (0.8 < κ ≤ 1)

*GRE* gradient echo

** Values in parentheses are 95% confidence intervals

**Table 3:** Intra-observer reliabilities between MRI sequences

Sequences		Kappa values (95% confidence interval)	*p* value
GRE axial vs. T2 axial	Reader 1	0.665 (0.57–0.76)	< 0.001
	Reader 2	0.657 (0.56–0.75)	< 0.001
GRE axial + T2 sagittal vs. T2 axial + T2 sagittal	Reader 1	0.979 (0.95–01.01)	< 0.001
	Reader 2	0.958 (0.92–1.00)	< 0.001

* Values are given as kappa (κ) values: poor (κ < 0.1), slight (0.1 ≤ κ ≤ 0.2), fair (0.2 < κ ≤ 0.4), moderate (0.4 < κ ≤ 0.6), substantial (0.6 < κ ≤ 0.8), and almost perfect (0.8 < κ ≤ 1)

** *GRE* gradient echo

CCSS grades had a weak to moderate correlation with clinical manifestations according to MRI sequences, with R ranging from 0.260 to 0.389 (Table 4). The GRE axial, GRE axial + T2 sagittal, and T2 axial + T2 sagittal images showed superior correlation (moderate) compared to the T2 axial image only (weak, Table 4) with clinical manifestations. There were no significant differences among GRE axial, GRE axial + T2 sagittal, and T2 axial + T2 sagittal images in the correlation between Kang grade and clinical manifestations.

**Table 4:** Correlation coefficients between stenosis grades and clinical manifestations according to MRI sequences

Sequences		Correlation coefficients	*P* value
GRE axial	Reader 1	0.363	< 0.001
	Reader 2	0.351	< 0.001
T2 axial	Reader 1	0.260	0.003
	Reader 2	0.273	0.002
GRE axial + T2 sagittal	Reader 1	0.389	< 0.001
	Reader 2	0.355	< 0.001
T2 axial + T2 sagittal	Reader 1	0.389	< 0.001
	Reader 2	0.348	< 0.001

* The level of correlation significance was 0.01

** The strength of the correlation ^®^ was characterized as follows: weak correlation (0.1 < *R* ≤ 0.3), moderate correlation (0.3 < *R* ≤ 0.7), relatively high correlation (0.7 < *R* ≤ 0.9), and very high correlation (0.9 < R)

*** *GRE* gradient echo

## Discussion

CCSS can result from a variety of causes and may lead to cord compression and disability. Existing image-based grading systems help us to communicate with clinicians and evaluate stenosis; among them, the Kang grading system correlates well with neurologic symptoms, and its inter-observer agreement has been reported to range from 0.768 to 0.925 [1, 4]. This can also be confirmed by our present study, which found that the inter-observer agreement was almost perfect (0.937–0.970) depending on the combination of MRI sequences (Table 2). Compared to the results of Lee et al. [6], the incidence rates of grades 2 and 3 were similar for any combination of image sequences, but the incidence of grade 0 was less than in previous studies (when comparing the results of radiologists with the same level of experience). We do not know the exact reason for the variation in these results, although participant age could be one cause; the mean age of 133 patients included in Lee et al.‘s study [6] was 44.1 years, while the mean age of 143 patients in this study was 51.7 years. Because disc degeneration and disc bulging progress with aging, partial obliteration of the subarachnoid space (grade 1 stenosis) becomes more prevalent in older populations, reducing the relative frequency of grade 0 findings. Our study revealed almost-perfect agreements between readers (κ > 0.93, Table 2), similar to the results of a previous study [6]. It can be assumed that, no matter what the MRI sequence is, the reader-to-reader reliability is consistently high, as radiologists are very familiar with Kang’s grading system and assign grades based on it. According to Table 3, the intra-observer reliabilities between the combined sequences (GRE axial + T2 sagittal vs. T2 axial + T2 sagittal) showed almost-perfect agreement, whereas the inter-observer reliabilities between the GRE axial and T2 axial images showed substantial agreement. The reason for this is probably due to differences in the assessment of cerebrospinal fluid (CSF) space between the spinal cord and vertebral body due to MRI physics differences. Fast spin-echo (FSE) sequences use refocusing pulses that preserve high signal intensity of static fluid, resulting in more uniform and conspicuous visualization of the pericordal CSF space. In contrast, gradient recalled echo (GRE) imaging lacks a refocusing pulse and is more sensitive to susceptibility and flow-related effects, which can lead to regional signal loss or signal heterogeneity within the CSF space on axial images. These differences may affect perceived degrees of subarachnoid space obliteration when axial images are interpreted in isolation. The GRE sequence can reduce the flow artifact, which could help in the evaluation of stenosis; however due to its low SNR, the resolution can be reduced, resulting in a false-positive result of compressive myelopathy. GRE imaging is less affected by phase-encoding ghost artifacts caused by pulsatile cerebrospinal fluid flow, which may improve delineation of the spinal canal in certain cases. However, because GRE sequences generally have lower signal-to-noise ratio and increased susceptibility sensitivity compared with FSE imaging, spatial resolution may be reduced in borderline cases, potentially leading to overestimation of stenosis severity rather than true compressive myelopathy. Fortunately, there were no cases of confusion between cases of grades 2 and 3 in this study. However, a more significant reason for the discrepancy in agreement lies in the fact that the Kang system is essentially an evaluation method that employs T2 sagittal images. Axial images are a secondary criterion. Although multilevel stenosis was excluded from this study, the imaging characteristics evaluated are not inherently level-specific. However, in cases of multilevel disease, reduced signal-to-noise ratio and cumulative artifacts may amplify discrepancies, particularly with GRE imaging.

Prior studies made recommendations for appropriate MR sequences in the evaluation of cervical degenerative changes [9–10]. Often FSE T2 axial image is combined with GRE axial image as each sequence has its own advantage and disadvantage. GRE axial image is superior in the differentiation of gray and white matter in the cord and has fewer CSF artifact [10]. Many clinical practices perform cervical spine MRI for evaluation of spinal stenosis. If GRE axial image is not inferior to FSE axial T2WI in the evaluation of the compressive myelopathy, GRE image can replace FSE T2WI.

In terms of clinical correlation, we found that it was inferior only to the T2 axial imaging (weak correlation, Table 4). The clinical correlations of the GRE axial image are similar to those of GRE axial with T2 sagittal imaging and those of T2 axial with T2 sagittal imaging (moderate correlation).

There are some limitations in our study. First, patients with multilevel cervical stenosis were excluded from the analysis. Because multilevel cervical stenosis is common in clinical practice, exclusion of these patients limits the generalizability of our findings. Second, in routine clinical practice, axial T2-weighted imaging is also used to evaluate spinal cord signal abnormalities, foraminal stenosis, and nerve root compression. Axial T2-weighted FSE images are often useful for detecting subtle spinal cord signal changes and foraminal stenosis. Because GRE imaging has different contrast characteristics and lower signal-to-noise ratio, replacing axial T2 FSE imaging could potentially reduce sensitivity for these findings. Therefore, complete replacement of axial T2 FSE imaging may not be appropriate in all clinical settings. Third, since this was a retrospective study, potential selection bias cannot be avoided. Fourth, this study was conducted at a single center, possibly limiting its generalizability. Fifth, as this study was a retrospective study, neurological symptoms could not be quantified; they were only classified as either positive or negative. This lack of accurate neurological sign evaluation may overestimate the actual accuracy of the Kang system. Sixth, our study lacks quantitative analyzes to assess spinal stenosis and spinal cord compression. Measurements including the surface area of the cord and ratio of the spinal canal to the spinal cord might better clarify the relationship between MRI findings and clinical manifestations. Seventh, only 3 cases underwent electromyography in this study and therefore this can be a limitation because several studies have shown inconsistent relationship between the degree of cervical spinal stenosis on MRI and the clinical manifestations.

In conclusion, the agreement of CCSS grading with axial GRE imaging and axial FSE T2WI was substantial. The correlation between MRI sequence and clinical manifestation was superior when using axial GRE images compared to axial FSE T2WI. Therefore, the axial GRE image can sufficiently replace the T2 axial FSE image in the evaluation of the CCSS.

## Data Availability

No datasets were generated or analysed during the current study.
